# Disease Progression and Phasic Changes in Gene Expression in a Mouse Model of Osteoarthritis

**DOI:** 10.1371/journal.pone.0054633

**Published:** 2013-01-28

**Authors:** Richard F. Loeser, Amy L. Olex, Margaret A. McNulty, Cathy S. Carlson, Michael Callahan, Cristin Ferguson, Jacquelyn S. Fetrow

**Affiliations:** 1 Department of Internal Medicine, Section of Molecular Medicine, Wake Forest University School of Medicine, Winston-Salem, North Carolina, United States of America; 2 Department of Computer Science, Wake Forest University, Winston-Salem, North Carolina, United States of America; 3 Department of Veterinary Population Medicine, College of Veterinary Medicine, University of Minnesota, St. Paul, Minneapolis, United States of America; 4 Department of Orthopedic Surgery, Wake Forest University School of Medicine, Winston-Salem, North Carolina, United States of America; 5 Department of Physics, Wake Forest University, Winston-Salem, North Carolina, United States of America; Cochin Hospital (AP-HP), and the University Paris Descarte, France

## Abstract

Osteoarthritis (OA) is the most common form of arthritis and has multiple risk factors including joint injury. The purpose of this study was to characterize the histologic development of OA in a mouse model where OA is induced by destabilization of the medial meniscus (DMM model) and to identify genes regulated during different stages of the disease, using RNA isolated from the joint “organ” and analyzed using microarrays. Histologic changes seen in OA, including articular cartilage lesions and osteophytes, were present in the medial tibial plateaus of the DMM knees beginning at the earliest (2 week) time point and became progressively more severe by 16 weeks. 427 probe sets (371 genes) from the microarrays passed consistency and significance filters. There was an initial up-regulation at 2 and 4 weeks of genes involved in morphogenesis, differentiation, and development, including growth factor and matrix genes, as well as transcription factors including Atf2, Creb3l1, and Erg. Most genes were off or down-regulated at 8 weeks with the most highly down-regulated genes involved in cell division and the cytoskeleton. Gene expression increased at 16 weeks, in particular extracellular matrix genes including Prelp, Col3a1 and fibromodulin. Immunostaining revealed the presence of these three proteins in cartilage and soft tissues including ligaments as well as in the fibrocartilage covering osteophytes. The results support a phasic development of OA with early matrix remodeling and transcriptional activity followed by a more quiescent period that is not maintained. This implies that the response to an OA intervention will depend on the timing of the intervention. The quiescent period at 8 weeks may be due to the maturation of the osteophytes which are thought to temporarily stabilize the joint.

## Introduction

Osteoarthritis (OA) affects over 27 million people in the United States and is similarly prevalent across the globe, making it the most common cause of chronic disability in adults [1], [2]. There are a number of well established risk factors for OA that include age, obesity, prior joint injury, genetics, joint anatomy, and occupational history related to joint use [3]. Current treatments for OA are limited and no treatment has been conclusively shown to alter disease progression. A better understanding of the biological factors which drive disease progression, particularly early-stage disease, is needed in order to develop more targeted therapies to stop or slow progression.

Studies of human OA are limited by its multifactorial nature, the lack of tissue that can be obtained at various stages of the disease and difficulties in defining disease onset. Basic mechanistic studies often are performed with tissue obtained at the time of joint replacement, which represents end-stage disease. These studies have focused largely on changes present in the articular cartilage; however, OA is a condition that affects the joint as an organ including not only the cartilage but also subchondral bone, synovium, ligaments and, in the knee, the meniscus (reviewed in [4]).

Various animal models of OA have been developed in order to study the disease process under more controlled conditions where disease onset and stages of progression can be better defined and evaluated. A commonly used model in mice is the destabilized medial meniscus (DMM) model where OA is surgically induced by transection of the medial meniscotibial ligament. In this model, OA results from altered joint biomechanics and the pathologic changes of cartilage destruction, subchondral bone thickening, and osteophyte formation are similar to the changes seen in human OA [5]. We recently used this model to compare the changes in OA severity and gene expression in young and older adult mice at a single time point of 8 weeks after DMM surgery [6]. The severity of OA changes in the joint of the older adult mice (12 months old at the time of surgery) was about twice that of the younger adult mice (12 weeks old at surgery) and significant differences were noted in the genes that were up and down-regulated at that time point.

The present study was designed as a time-course experiment to evaluate changes in gene expression during the development of OA and to compare these changes to the histologic progression of the disease. The recently reported comparison of gene expression at the 8 week time point in young adult and older adult mice [6] was a sub-study of the present work, but because of the lack of sufficient numbers of older animals the time course study only includes the young adult mice. Similar to the previous report, the histological assessment and the gene microarray analysis were designed in a manner which considered OA as a disease of the joint as an organ and, thus, were not limited to a single tissue within the joint. The general hypothesis was that different stages of the disease process would be characterized by unique gene expression signatures. Knowledge of the biological processes regulated by these genes could provide new information about the OA disease process. The results demonstrated progressive histological changes of OA beginning within two weeks of DMM surgery and progressing over the 16 week time course. These were accompanied by phasic changes in gene expression, with the peak number of up-regulated genes found at 4 weeks after induction of OA, a significant decline in expression at 8 weeks, and an increase again at 16 weeks with different clusters of genes being more prominent at each time point.

## Materials and Methods

### Animals and surgical induction of OA

Male C57BL/6 mice (n = 129) were purchased from Charles River Inc. and were 12 weeks of age at the time of DMM surgery or sham surgery performed as a control. Animal use was carried out in strict accordance with the recommendations in the Guide for the Care and Use of Laboratory Animals of the National Institutes of Health. The protocol was approved by the Wake Forest School of Medicine Animal Care and Use Committee (protocol #A09-622). All surgery was performed under isoflurane anesthesia, and all efforts were made to minimize suffering. Butorphanol (2.5mg/kg) was administered in the event of pain.

Details of animal housing and induction of OA have been recently published [6]. Mice were operated on in waves and at the time of surgery were randomly assigned to a surgical group and termination time point so that the date when the surgery was performed would not influence the results. For the present study, a group of 9 mice was used for collection of RNA at time 0 (before surgery) when the animals were 12 weeks old. For the other time points, 6 DMM and 6 sham controls were sacrificed at 2, 4, 8, and 16 weeks after surgery for histological analysis and 9 DMM and 9 sham controls were sacrificed at each time point for RNA isolation. As noted above, the histological sections and RNA used for the 8 week time point were from the same samples as those previously reported [6] but here were reanalyzed as part of the time course study.

### Histological analysis

Hind limbs from both the operated side and the contralateral side of each animal were dissected and processed for histological analysis. The details of fixation, processing, and sectioning, as well as the histological analysis, were as recently published [6], [7]. In brief, hematoxylin and eosin and safranin-O mid-coronal sections of each stifle joint were obtained and representative sections from each joint graded by an evaluator (MAM) blinded to group assignment. The evaluation included the Articular Cartilage Structure (ACS) score (0–12), Safranin-O (Saf-O) staining score (0–12), articular cartilage thickness and area,% chondrocyte cell death, subchondral bone area and thickness, and osteophyte total area. Because the most severe changes were consistently found on the tibial side of the joints, the analysis was focused on the tibial plateaus. Synovitis is only apparent in severe disease in this model and given the lack of sufficient numbers of severe samples there was no attempt to score synovial changes.

Immunohistochemistry was performed on sections from 3 DMM and 4 sham control mice at the 16-week time point in order to examine tissue distribution of type III collagen, fibromodulin and proline-arginine-rich end leucine-rich repeat protein (Prelp). Antigen retrieval and blocking steps were as recently described [6]. Primary antibodies were acquired from Abcam Inc (Cambridge, MA) and included rabbit polyclonal antibodies to Collagen III (ab7778), Prelp (ab103868), and fibromodulin (ab81443). Secondary antibodies and detection were as described [6]. Immunostained sections were graded for cell and matrix staining using a scale of no immunopositivity (−) to marked immunopositivity (++++).

### RNA preparation and microarray analysis

Knee joint tissue from the medial joint compartment was obtained from the time 0 group and the operated hind limbs from the other time points and processed for RNA isolation as described [6]. The tissue included tibial plateau and femoral condyle articular cartilage, subchondral bone with any osteophytes, meniscus, and the joint capsule with synovium. The tissue was treated with RNAlater® (Invitrogen) prior to freezing and storage at −80°C. RNA was extracted by homogenization using the Precellys 24 tissue homogenizer (Bertin Technologies purchased from MO BIO) as described [6] and the amount and quality of the RNA was determined using an Agilent 2100 Bioanalyzer.

RNA was pooled prior to microarray analysis such that 3 randomly selected samples from each surgical group and time point were pooled to create a each biological replicate. Because 9 mice were used for each experimental group, a total of three biological replicates per group were analyzed using the Affymetrix Mouse Genome 430 2.0 oligonucleotide arrays as described [6]. One replicate pool, which was from week two DMM mice, did not meet the RNA integrity level needed for microarray analysis; thus, this pool was not analyzed further, leaving two pools for the week two DMM mice.

### Normalization and processing of microarray data

Microarrays were imaged and the resulting data normalized using systematic variation normalization (SVN) as previously described [6], [8]. Reported data included the log_2_ of the signal intensity and the Affymetrix detection p-value for each probe set. Because some genes had more than one probe set, we provide results for both numbers of genes and probe sets when the numbers differed. The complete dataset has been provided to the Gene Expression Omnibus (GEO) repository (accession number GSE41342). Relative gene expression in the samples collected from the sham-operated control animals was calculated to identify those genes that changed as the animals aged over the 16 week time course independent of DMM-induced OA. For this analysis, sham gene expression was calculated as the signal log ratio (SLR) of the average time 0 intensity (baseline un-operated controls) to each sham replicate pool for each time point. The average of the time 0 intensity was used to ensure a consistent baseline, and each biological replicate of the sham arrays was analyzed individually to allow biological consistency to be measured in the analysis. The SLR is the log_2_ of the fold change and so an SLR of 0.5 =  1.4-fold. After SLR calculations, each probe set had 12 associated SLR values (3 for each time point—2, 4, 8 and 16 weeks), which together are referred to as the “sham time course”.

Relative gene expression changes for the DMM time course were calculated to identify those genes that changed over time due to the DMM-induced changes in the joints. For this analysis, the sham results were used as a time-matched control (sham 2 weeks used as control for DMM 2 weeks etc.). Sham replicate signal intensities for each time point were averaged to ensure a consistent baseline for each replicate. DMM gene expression was then calculated as the time-matched signal log ratio of the average sham intensity to each DMM replicate for each time point, where each DMM replicate was analyzed individually. After SLR calculations, each probe set had 11 associated SLR values (2 for the 2 week time point, and 3 each for 4, 8 and 16 weeks), which are referred to as the “DMM time course”.

### Filtering and clustering of microarray data

**Figure 1:**
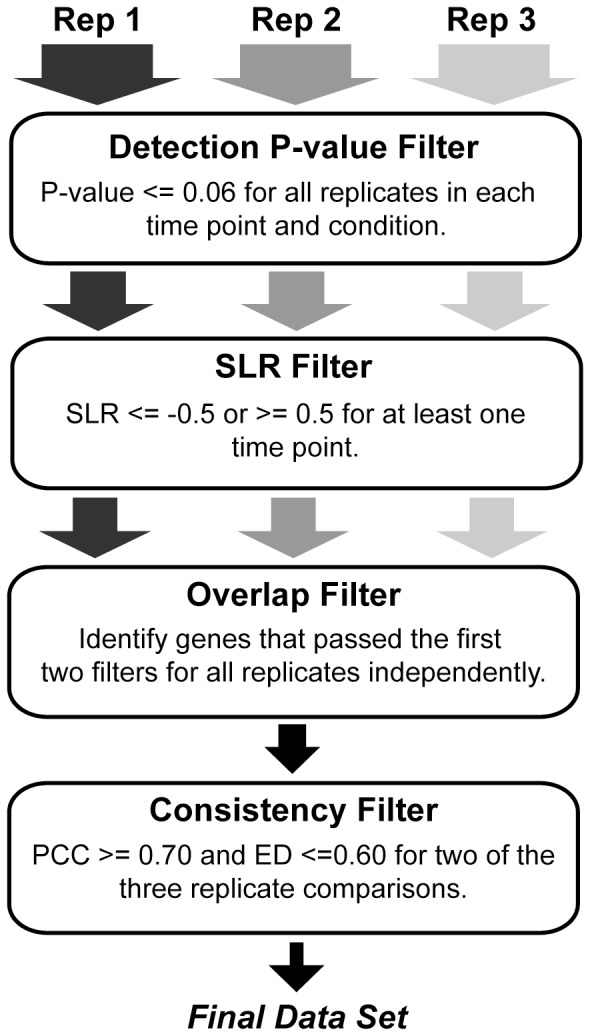
Flow chart of filtering process. After data normalization, SLR values were calculated as described in the methods, and replicate pools were randomly paired to obtain 3 complete time courses—referred to as Rep 1, Rep 2 and Rep 3. The first 2 filtering steps were done for each replicate independently, and identified probe sets that were significantly detected on the chip (detection p-value filter; all p-values < = 0.06) as well as significantly differentially expressed (SLR filter; SLR was < = −0.5 or > = 0.5 for any one time point). The overlap filter intersected all three filtered lists to identify the set of probe sets that passed the first 2 filtering steps independently in all replicates. Finally, probe sets were filtered for consistency in pattern (PCC score) and magnitude (ED score) of expression over time across all replicate time courses (consistency filter), where 2 of the 3 PCC scores had to be > = 0.70, and 2 of the 3 ED scores had to be < = 0.60. Those probe sets passing all filtering criteria were considered consistently and significantly differentially expressed across the time course in all 3 biological replicates, and were used for further analysis. Results of this process on each of the data sets described in the text can be found in Tables S1 and S2.

Relative gene expression for the sham time course and DMM time course was filtered for differentially expressed genes with consistent expression changes over time as previously described [9] with a few alterations. Briefly, both data sets were filtered in a step-wise fashion as illustrated in **Figure 1**. Replicate time courses were filtered individually in the first two steps to identify those transcripts that were significantly detected on the chip over background (detection p-value < = 0.06 for all time points and control), and for those that were differentially expressed in at least one time point (SLR ≥0.5 or ≤−0.5 which converts to ≥1.4-fold and ≤−1.4-fold). These data sets were intersected to identify those probe sets that passed the first two filtering steps independently in all replicates. Finally, transcripts were filtered for consistency of expression pattern (Pearson’s correlation; PCC) and magnitude (Euclidean distance; ED) over time. An all-by-all comparison was done for each replicate time course profile resulting in 3 PCC and 3 ED scores for each probe set. Requiring all 6 scores to pass selected criteria was found to be too strict (data not shown). Therefore, only 2 of the 3 PCC and ED scores were required to pass selected cutoffs: PCC > = 0.70 (a standard statistical threshold for positive correlation) and ED < = 0.60 or < = 0.81 for the DMM and sham time courses, respectively (the median ED score for each data set). Additionally, due to the missing 2-week DMM data for one of the replicates, all PCC and ED comparisons with this time course were done using only the 4-, 8- and 16-week time points. Probe sets passing all filtering criteria were considered to have been consistently and significantly expressed.

Filtered DMM time course gene expression data were clustered using the updated consensus clustering option provided by version 3 of SC^2^ATmd [10]—sham time course data was not clustered in this work. The option to perform consensus clustering using a threshold to identify genes that cluster together less than 100% of the time was added to SC^2^ATmd’s functionality since its original release. This was implemented by converting the consensus matrix to an adjacency matrix and then using MATLAB’s depth first search algorithm to identify consensus clusters. A figure of merit (FOM) analysis was run to determine the optimal number of clusters present in each replicate data set and to identify which clustering algorithm formed the most homogeneous clusters with respect to Euclidean distance [9], [11]. As determined by the FOM analysis, consensus clustering was run using 10 starting clusters with k-means as the clustering algorithm and Euclidean distance as the distance metric. Each replicate data set was clustered 10 times, and a consensus over all 30 runs was calculated. Performing more than 10 iterations on each replicate did not significantly improve cluster homogeneity (data not shown). Probe sets clustering together 83% of the time were identified as a consensus cluster. Increasing or decreasing this threshold resulted in small clusters with many singletons or large clusters that were uninformative, respectively; thus, the 83% threshold was chosen as an acceptable balance between these two extremes

### Annotation analysis

Annotation enrichment analysis was performed using the Database for Annotation, Visualization, Integration and Discovery (DAVID) [12], [13]. The Functional Annotation Clustering tool provided by DAVID was run using the Affymetrix Mouse 430 2.0 chip as background, with all other options left at their default settings. Annotations of clusters were considered significant if the Enrichment Score was greater than or equal to 1.3 (equivalent to a p-value < = 0.05).

### Real-time PCR

Real-time PCR was performed using samples of RNA from the same pools used for the microarrays as previously described [6] using the SYBR®Green-based method with optimized primer sets obtained in the RT2 qPCR Primer Assays (SABiosciences/Qiagen) and with TATA box-binding protein (TBP) used to normalize relative expression.

### Statistical analysis

Analysis of the microarray data is presented above. The histological data and the real time PCR data were found to be normally distributed. Histological data from both the lateral and medial tibial plateaus was evaluated using ANOVAs (intra-animal comparisons) and repeated measures analyses (inter-animal comparisons) using SPSS version 17.0 (IBM Corp, Somers, NY). The real time PCR results at each individual time point for sham controls and DMM animals were analyzed using t tests with StatView 5.0 software (SAS, Cary, NC).

## Results

### Histologic progression of joint tissue changes induced by DMM surgery

**Figure 2:**
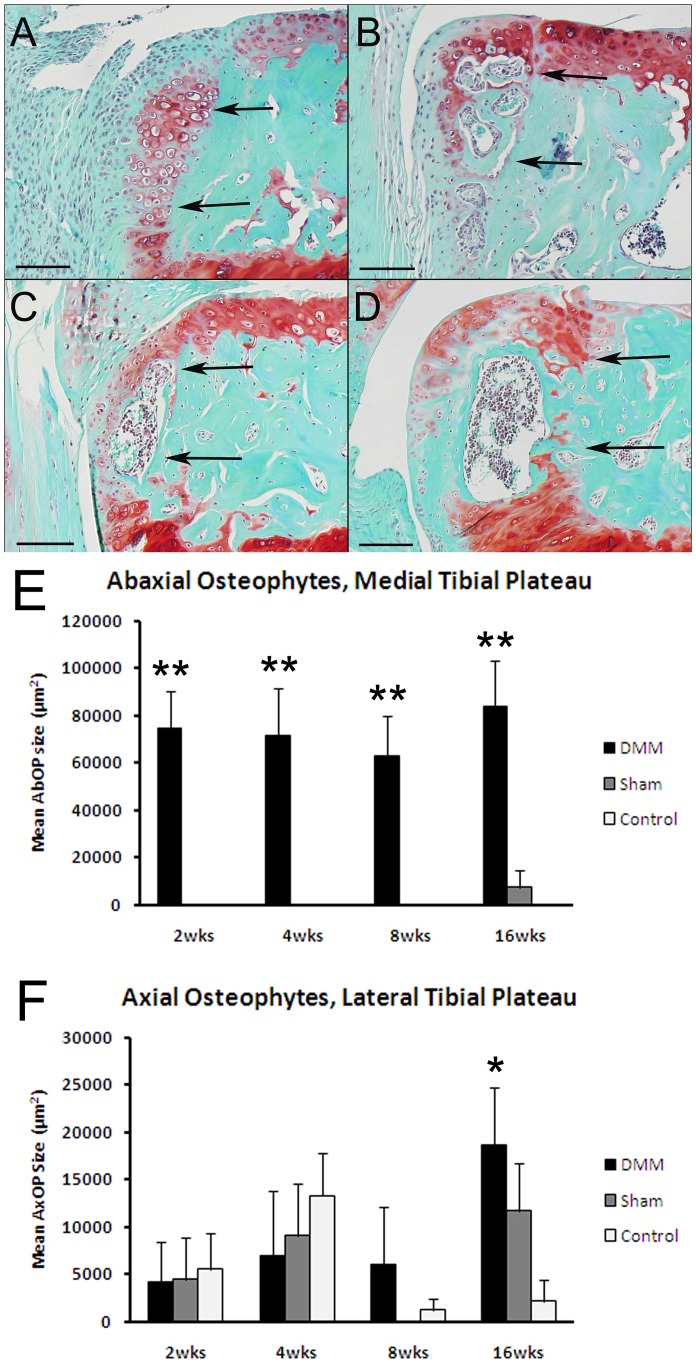
Histology results demonstrating osteophyte formation over the time course. Sections from DMM joints obtained 2 weeks (A), 4 weeks (B), 8 weeks (C), and 16 weeks (D) after DMM surgery were stained with safranin-O. Bar  = 100 µm. Arrows point to the original medial abaxial joint margin and abaxial osteophytes that formed at this site. These start as chondrophytes at 2 weeks (A) and progressively mature. Histomorphometric measures included the size of the abaxial osteophytes on the medial plateau (E) and axial osteophytes on the lateral plateau (F) for the DMM, sham, and contralateral control groups as indicated. **p<0.0001 vs sham and control; *p = 0.02 vs sham and control by ANOVA.

The sham operated joints and the contralateral control (non-operated joints from sham and DMM animals) demonstrated minimal to no pathological changes over the 16 week time course. Among the earliest changes observed in the DMM joints at 2 weeks after surgery were abaxial osteophytes in the medial tibial plateau. Osteophytes were primarily cartilaginous at 2 weeks, were ossified at 4 weeks, and were composed of trabecular bone that contained marrow spaces occupied by hematopoietic tissue at 8 and 16 weeks (**Figure 2A, B, C, D**). Interestingly, the cross-sectional area of the osteophytes did not change significantly from 2–16 weeks (**Figure 2E**). Much smaller and more variable axial osteophytes were noted on the lateral tibial plateaus but, unlike the medial abaxial osteophytes, their size was not significantly different among the three groups until 16 weeks after surgery, when they were significantly larger in the DMM relative to the sham and contralateral control joints (**Figure 2F**).

**Figure 3:**
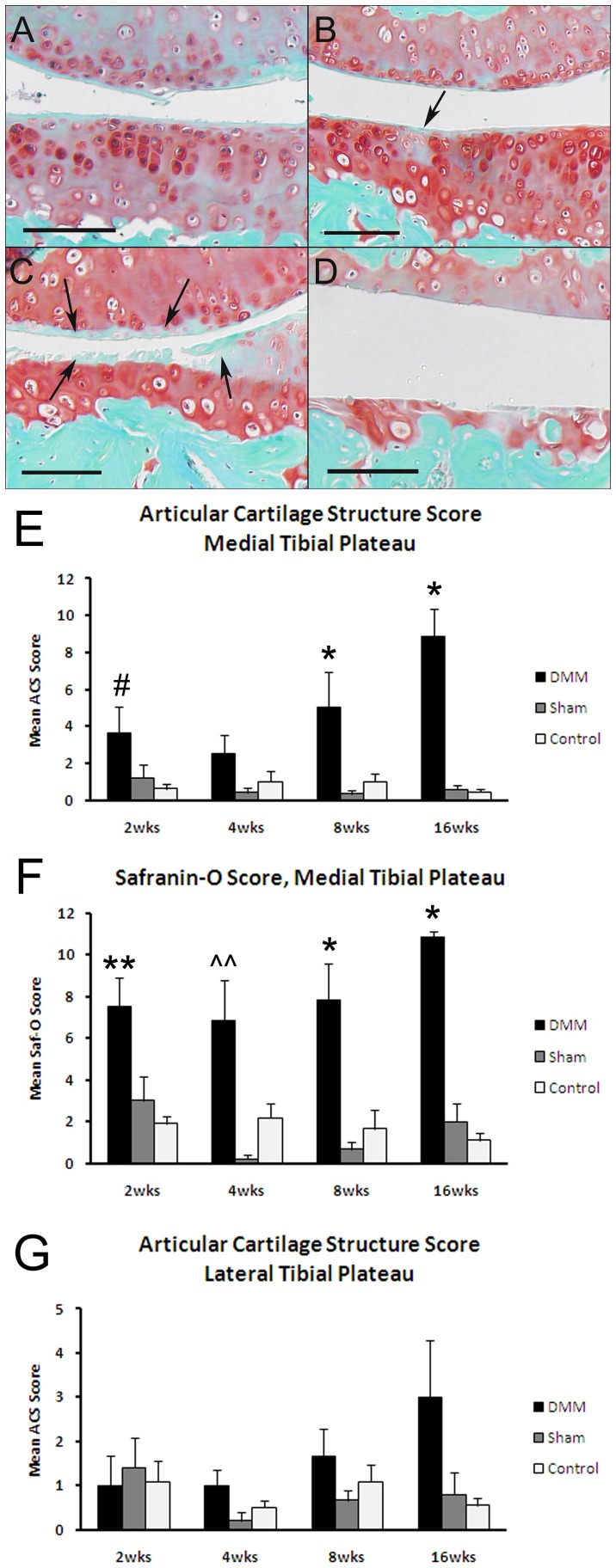
Histology results demonstrating articular cartilage lesions over the time course. Sections from DMM joints obtained 2 weeks (A), 4 weeks (B), 8 weeks (C), and 16 weeks (D) after DMM surgery were stained with safranin-O. Bar  = 100 µm. Arrows indicate an early cartilage lesion in the medial tibial plateau at 4 weeks (B) and a more severe lesion at 8 weeks (C) on the medial tibial plateau (lower tissue in image) and the femoral condyle (upper tissue in image). Note advanced articular cartilage loss at 16 weeks. Articular cartilage structure scores (E) and safranin-O scores (F) for the medial tibial plateaus and articular cartilage structure scores for the lateral tibial plateau (G) are shown for the DMM, sham, and contralateral control groups as indicated. *p<0.05 vs sham and control; ??p = 0.01 vs sham; **p<0.01 vs control; #p<0.05 vs control by ANOVA.

There were mild articular cartilage lesions noted at 2 weeks in the medial tibial plateaus of the DMM joints, resulting in an increase in the articular cartilage structure score and an increase in the Saf-O scores relative to sham and contralateral control joints at this time point (**Figure 3**). The articular cartilage structure scores increased progressively over the 16 weeks in the DMM joints while the Saf-O scores remained more stable over time. At 16 weeks, there was a mild increase in the articular cartilage structure score at the lateral tibial plateau in the DMM joints but the mean score was less than half of that seen in the medial tibial plateau (**Figure 3G**). No significant differences were seen among the groups or time points for Saf-O scores in the lateral tibial plateaus (data not shown). In addition, there were no significant differences in articular cartilage thickness over the course of the study in any of the groups (data not shown).

**Figure 4:**
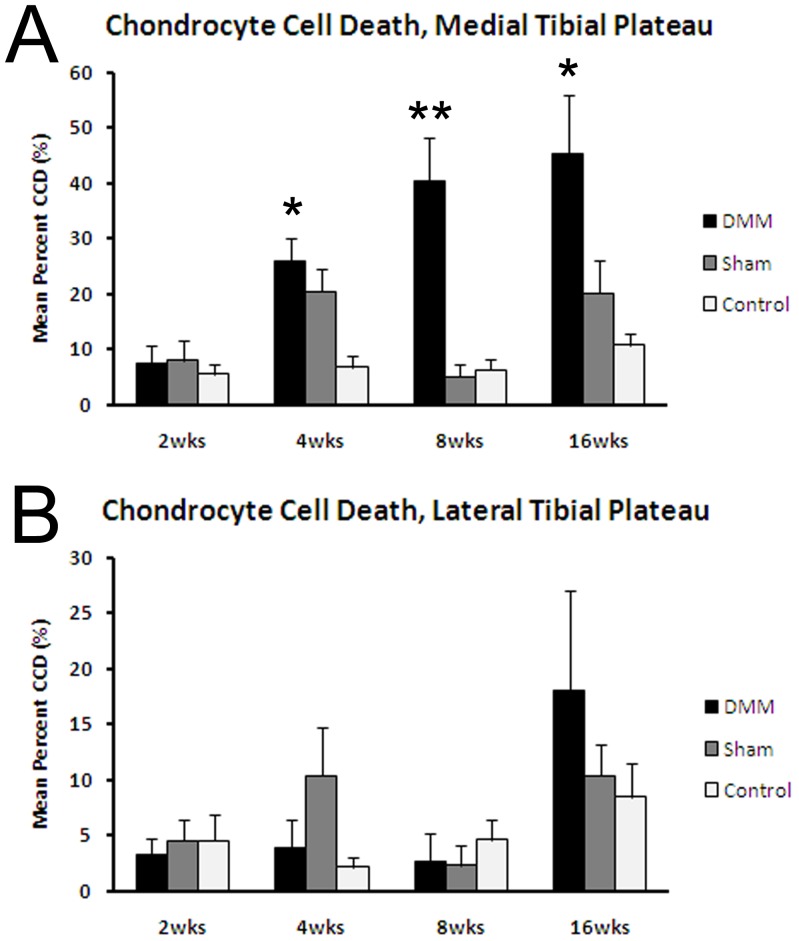
Histologic analysis of chondrocyte death over the time course. Dead chondrocytes were counted on H&E sections from the medial tibial plateau (A) and lateral tibial plateau (B) from DMM, sham, and contralateral control joints at the various time points after surgery. *p<0.05 vs sham and control joints; **p<0.001 vs sham and control joints by ANOVA.

Chondrocyte death in the medial tibial plateau articular cartilage was significantly increased at 4 weeks in the DMM and sham joints relative to the non-operated controls, and increased further in the DMM joints at 8 and 16 weeks (**Figure 4A**). Dead chondrocytes also were apparent in the lateral tibial plateaus at week 16 but the area occupied by dead chondrocytes was less than half that in the medial tibial plateau, nor was there a difference in this parameter among the surgical groups at any time point (**Figure 4B**). We have recently published evidence for chondrocyte death in the DMM model at 8 weeks after surgery including findings of positive TUNEL staining consistent with apoptosis [14]; thus, the mechanism of cell death was not pursued in the present study.

The subchondral bone was thicker in the medial tibial plateau than the lateral tibial plateau in all three groups and at all time points (**Table 1**). At all time points, the subchondral bone thickness and area were larger in the medial tibial plateau of the DMM group than in the other two groups, although this difference was only significant at the 4-week time point. In all groups, there was a gradual increase in subchondral bone thickness and area over the 16 weeks of the study.

**Table 1:** Histomorphometric measures of subchondral bone area and thickness in DMM and sham control joints.

	Time	Treatment Group	Medial Tibial Plateau	ANOVA	Lateral Tibial Plateau	ANOVA
**SCB Area**	2 week	DMM	85777 (±12232)	NS	42703 (±5768)	NS
**SCB Area**	2 week	Sham	66140 (±8668)	NS	46342 (±6410)	NS
**SCB Area**	2 week	Control	70557 (±5895)	NS	48279 (±4694)	NS
**SCB Area**	4 week	DMM	116943 (±18235)	p = 0.011	67074 (±5367)	NS
**SCB Area**	4 week	Sham	71067 (±11028)	p = 0.011	57426 (±8690)	NS
**SCB Area**	4 week	Control	71758 (±5284)	p = 0.011	55541 (±5293)	NS
**SCB Area**	8 week	DMM	147622 (±13141)	NS	50446 (±8643)	NS
**SCB Area**	8 week	Sham	122046 (±19956)	NS	68432 (±11352)	NS
**SCB Area**	8 week	Control	103391 (±9112)	NS	62735 (±5925)	NS
**SCB Area**	16 week	DMM	144786 (±21706)	NS	75524 (±16120)	NS
**SCB Area**	16 week	Sham	124481 (±23749)	NS	62240 (±10563)	NS
**SCB Area**	16 week	Control	116784 (±15843)	NS	66090 (±7798)	NS
**SCB Thickness**	2 week	DMM	74.1 (±9.4)	NS	40.5 (±4.6)	NS
**SCB Thickness**	2 week	Sham	58.7 (±6.2)	NS	45.7 (±5.7)	NS
**SCB Thickness**	2 week	Control	63.3 (±5.3)	NS	45.5 (±4.1)	NS
**SCB Thickness**	4 week	DMM	99.2 (±13.4)	p = 0.011	56.3 (±4.0)	NS
**SCB Thickness**	4 week	Sham	64.2 (±9.8)	p = 0.011	51.9 (±7.8)	NS
**SCB Thickness**	4 week	Control	63.5 (±4.3)	p = 0.011	49.8 (±4.1)	NS
**SCB Thickness**	8 week	DMM	120.8 (±9.9)	NS	48.4 (±5.1)	NS
**SCB Thickness**	8 week	Sham	99.4 (±11.3)	NS	69.0 (±11.7)	NS
**SCB Thickness**	8 week	Control	90.8 (±7.2)	NS	57.0 (±4.7)	NS
**SCB Thickness**	16 week	DMM	111.9 (±20.4)	NS	66.2 (±9.8)	NS
**SCB Thickness**	16 week	Sham	106.8 (±15.7)	NS	60.1 (±7.9)	NS
**SCB Thickness**	16 week	Control	101.2 (±12.6)	NS	60.1 (±6.2)	NS

Subchondral bone (SCB) area ( µm^2^) and thickness ( µm) were measured as detailed in the Materials and Methods. The p values are the ANOVA values between the treatment groups within each time point and the significance noted was between the DMM vs sham and contralateral controls. For each group and each time point sections from an n of 6 animals were measured.

### Changes in gene expression over time in the sham control joints

**Figure 5:**
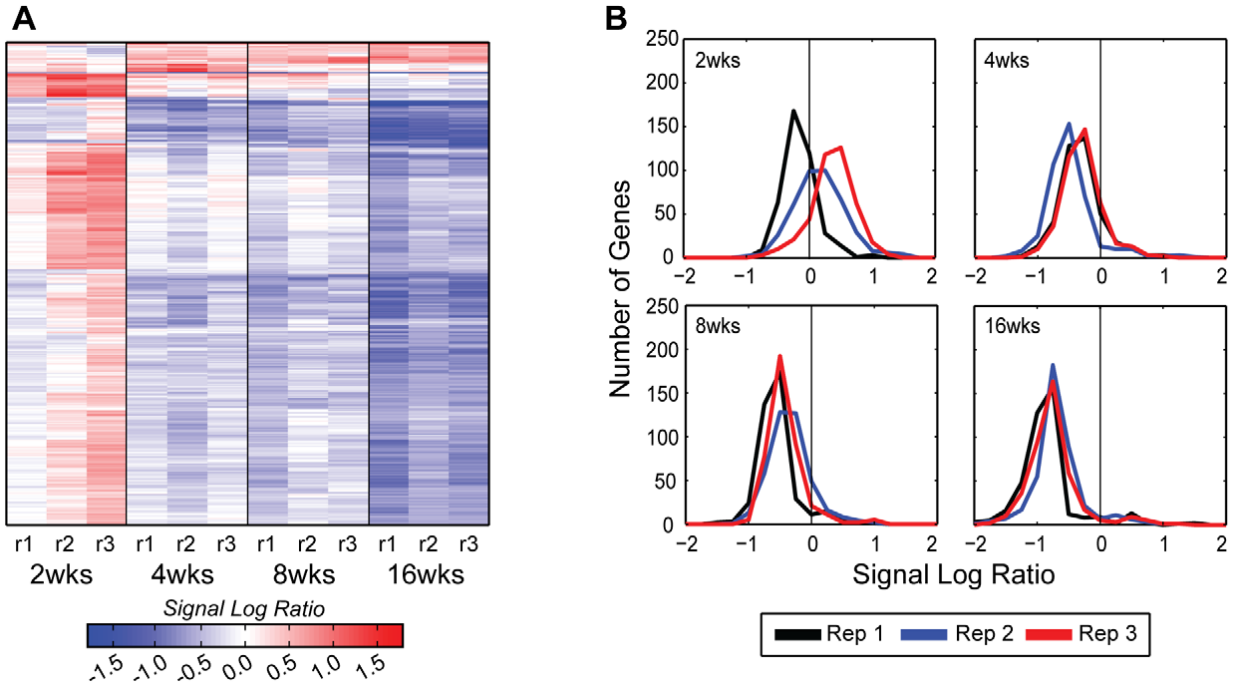
Global view of the differentially expressed genes in the sham time course. A) A heat map illustrates that the majority of differentially expressed genes (406 probe sets for 347 genes), in the sham time course are down-regulated, with few being up-regulated over time. Heat map rows represent genes, and columns represent replicates of each time points. Red indicates up-regulation, blue down-regulation and white indicates no change in gene expression from the respective control (see Methods). B) Gene expression distributions of the sham time course exhibit a uni-modal distribution throughout the experiment; however, the distributions center becomes shifted toward the negative side of zero at 4 weeks, indicating a global down-regulation of these genes over time due to aging. An SLR of zero is marked by a vertical line on the graph, and replicate time courses are colored as follows: replicate 1 =  black, replicate 2 =  blue, replicate 3 =  red.

To detect changes in gene expression occurring over the 16 week time period of the study that could be due to aging of the animals, we analyzed the gene expression results in the sham joints relative to the time 0 baseline. Filtering of the data revealed 347 genes (406 probe sets) significantly and consistently regulated over time in the sham joints. A complete list of these genes is provided in File S1. The 2-week time point had the largest number of up-regulated genes and this was followed by a progressive decrease in expression of the majority of genes over the 16 week time course (**Figure 5A and 5B**). The set of 347 genes was analyzed by DAVID’s Functional Annotation Clustering tool [12], which identified 36 groups of significantly over-represented annotations with enrichment scores ≥1.3 (File S1). Most of the annotations were related to signal peptide, extracellular matrix, development, differentiation and morphogenesis, consistent with a maturation process occurring over the 16 week time course.

A small subset of 25 genes remained up-regulated over the time course (File S1). Functional Annotation Clustering was performed on this subset of genes, and only one group of significantly over-represented annotations was identified. This group of terms included plasma membrane, glycoprotein, immune response, and cell surface linked signal transduction terms.

### Gene expression in DMM joints relative to sham controls

**Figure 6:**
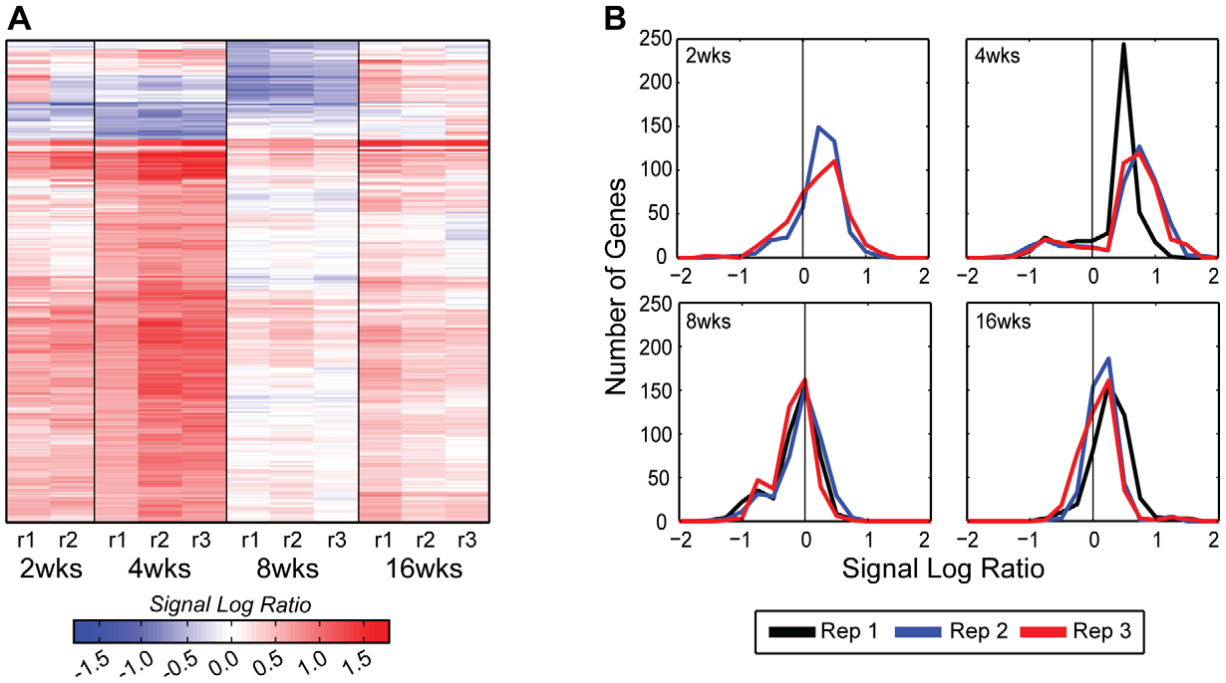
Global view of the differentially expressed genes in the DMM time course. A) The heat map illustrates that the majority of differentially expressed genes (427 probe sets for 371 genes) were up-regulated throughout the DMM time course, except for a small group of genes, which were down-regulated 4 and 8 weeks after surgery (see Figure 5A legend for heat map formatting details). B) The DMM time course exhibits a bi-modal distribution at 4 and 8 weeks after surgery, with the majority of genes being up-regulated at 4 weeks and not different from control at 8 weeks (see Figure 5B legend for formatting details).

The time course results for changes in expression in the DMM joints relative to time-matched sham joints revealed 371 genes (427 probe sets) that were significantly and consistently regulated over the three biological replicates (File S2). Examination of a heat map of these genes as well as the SLR distributions (**Figure 6A and 6B**) reveals a phasic pattern. The majority of genes were up-regulated at 2 and 4 weeks followed by reduced expression or down-regulation at 8 weeks and then some increase in expression at 16 weeks although not to the level of weeks 2 and 4.

**Figure 7:**
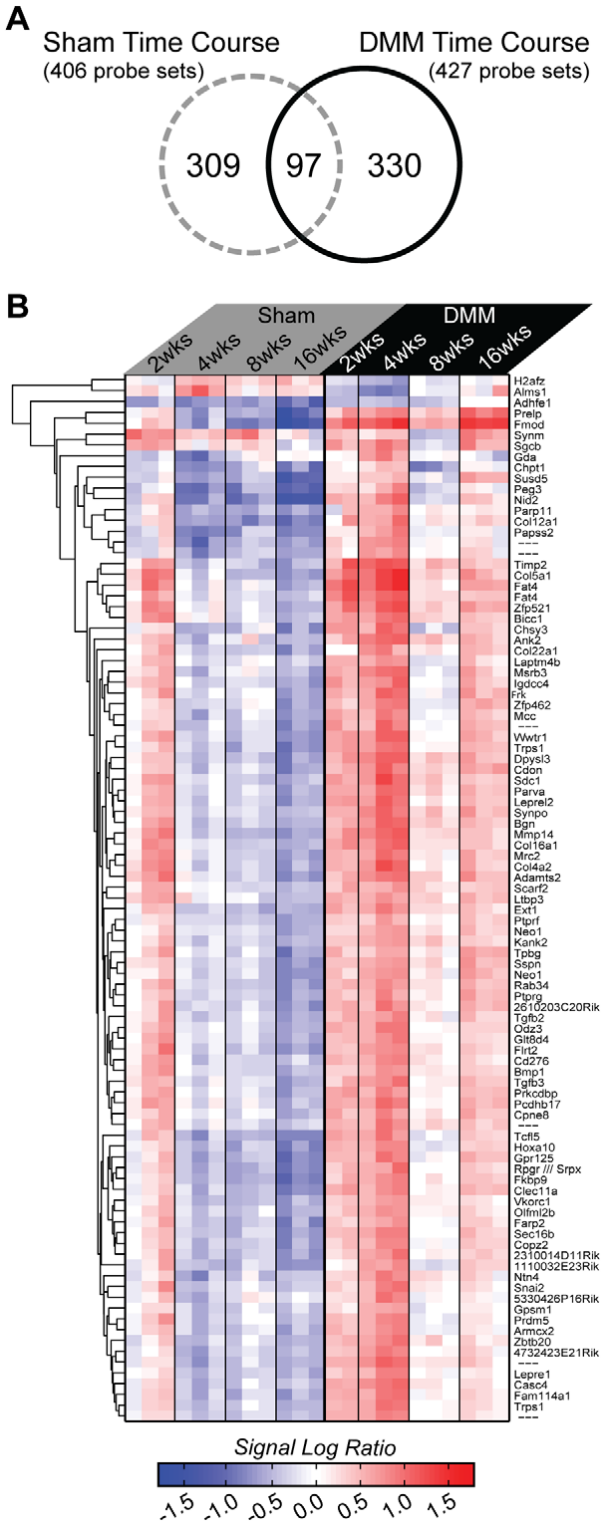
The group of genes differentially expressed in both sham and DMM data sets. Intersection of the 406 differentially expressed sham time course probe sets and the 427 DMM time course probe sets revealed 97 probe sets (94 genes) found in both data sets. A) A Venn diagram illustrates the number of probe sets in each data set as well as the overlap, and indicates that the majority of genes with OA induced expression changes are not altered in sham joints. B) A heat map of gene expression changes for the 97 intersecting probe sets was created using hierarchical agglomerative clustering and Euclidean distance, with sham expression on the left and DMM expression on the right. The heat map is formatted as in Figures 5A and 6A with the addition of the hierarchical dendrogram on the left and the gene names on the right.

These results were much different from the time course results noted in the sham joints, consistent with changes in gene expression due to induction of OA by the DMM surgery. An overlap analysis revealed 94 genes (97 probe sets) that were in common in the DMM/sham and sham/baseline control datasets (**Figure 7A** and File S3). Many of these were extracellular matrix genes including Prelp, fibromodulin, several collagens (Types IV, V, XII, XVI, and XXII), and biglycan. A heat map revealed that almost all of these genes displayed very different expression profiles. Most were down-regulated over time in the sham controls but up-regulated in the DMM joints, particularly at 2 and 4 weeks (**Figure 7B**). Of the 94 genes in common between the DMM and sham joints, almost all were up-regulated in the DMM joint, with the exception of three: H2afz (predicted gene 6722///H2A histone family, member Z), Alms1(Alstrom syndrome 1), and Adhfe1(iron-containing alcohol dehydrogenase), which were down-regulated in the DMM joints. These genes do not have any known role in joint development or in OA.

The 94 genes in common between DMM/sham and sham/baseline were analyzed by DAVID. This returned 10 significant annotation groups (File S3) that included terms such as extracellular matrix, cell adhesion, collagen, skeletal development, EGF-like domains, fibronectin, leucine rich repeats, and endoplasmic reticulum.

### Consensus clustering of the genes regulated during the development of OA in the DMM joints

**Figure 8:**
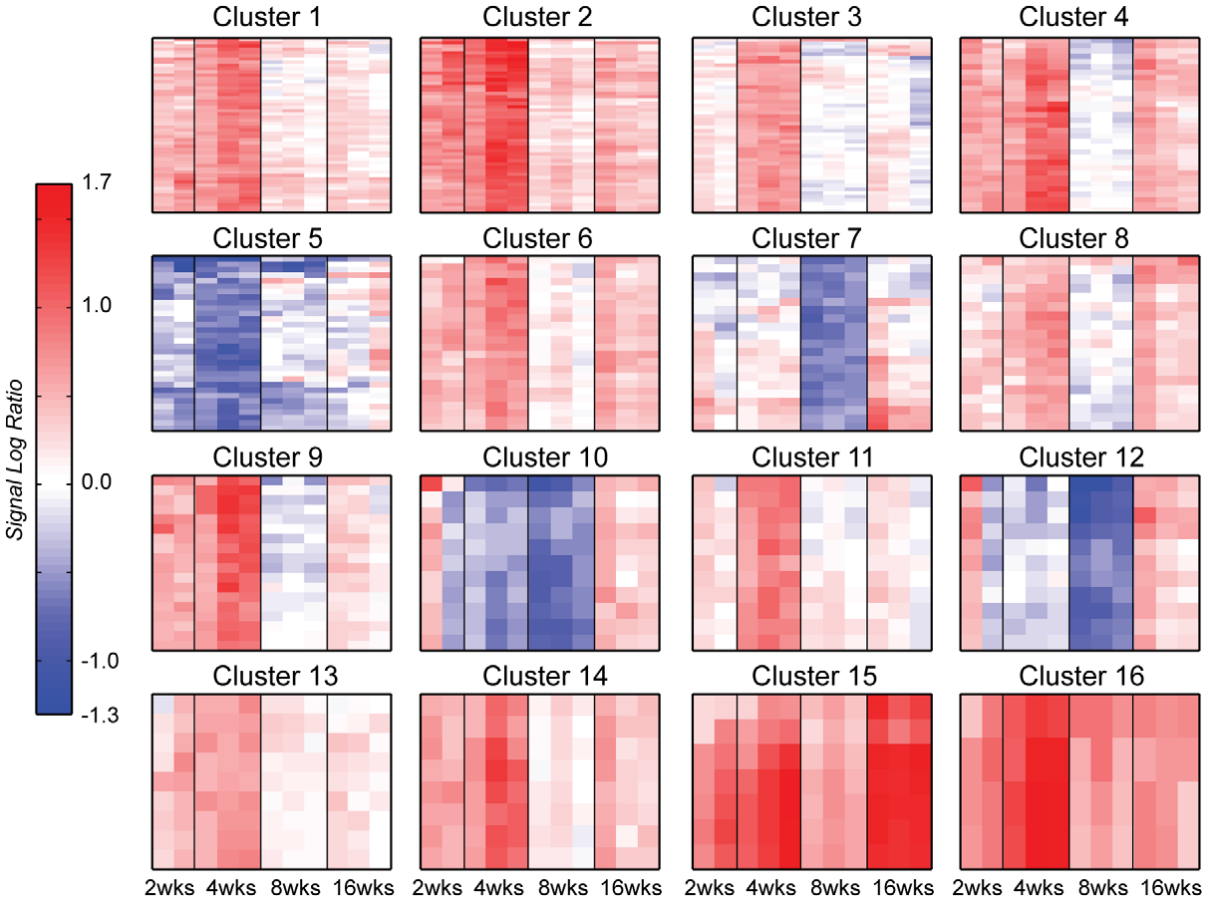
Heat maps of the largest 16 consensus clusters in the DMM time course. Rows represent genes and columns are time point replicates in the following order: 2 weeks rep2, rep3; 4-, 8- and 16 weeks rep1, rep2, rep3. Red indicates up-regulation, blue down-regulation and white indicates no change in expression from the sham time-matched control. All heat maps in this figure are colored on the same scale for easy comparison with maximum up-regulation at 1.7 SLR and maximum down-regulation at −1.3 SLR. A variety of expression patterns are present with most including significant up-regulation at 4 weeks. Only four clusters contain down-regulated genes (clusters 5, 7, 10 and 12), and cluster 15 is the only one with significant up-regulation 16 weeks after surgery.

Consensus clustering of genes with altered expression after DMM surgery identified 27 clusters with 2 or more probe sets and 38 genes (39 probe sets) that were classified as outliers (singletons). The complete list of the genes found in each cluster and the DAVID analysis for the clusters are found in File S2. Heat maps of the largest 16 clusters are shown in **Figure 8** and reveal striking differences in the temporal patterns of gene expression over the 16 week time course. The significant annotations for these clusters from DAVID analysis are shown in **Table 2**.

**Table 2:** Significant DAVID annotations for the top 16 Gene Clusters.

Cluster	Significant DAVID Annotations
1	Embryonic organ morphogenesis, inner ear morphogenesis, embryonic organ development, positive regulation of cell differentiation, positive regulation of developmental process, regulation of cell proliferation
2	Collagen, signal peptide, disulfide bond, glycoprotein, extracellular region, extracellular matrix, cell adhesion, glycosaminoglycan binding
3	Regulation of transcription, DNA binding, regulation of RNA metabolic process, Zinc finger C2H2-like, transition metal ion binding
4	None significant
5	None significant
6	Signal, signal peptide, glycoprotein, glycosylation site:N-linked, disulfide bond, cell adhesion, phosphorus, metabolic process, transmembrane receptor protein serine/threonine kinase signaling pathway, skeletal system development/morphogenesis, enzyme linked receptor protein signaling pathway, protein amino acid phosphorylation, growth factor, growth factor activity, regulation of cell proliferation
7	Mitotic cell cycle, cell cycle phase/process, nuclear/cell division, organelle fission, cytoskeletal protein binding, intracellular non-membrane-bounded organelle,
8	Cell adhesion, biological adhesion
9	None significant
10	None significant
11	None significant
12	Carbohydrate metabolism, glucose/hexose/monosaccharide metabolic process
13	None significant
14	None significant
15	Extracellular matrix, secreted, disulfide bond, signal peptide, glycoprotein
16	Extracellular matrix, secreted, disulfide bond, signal peptide, glycosylation site:N-linked, glycoprotein

Only clusters 5, 7, 10 and 12 exhibited significant down-regulation at specific time points. Cluster 5 contained genes down-regulated at week 4; cluster 10, genes down-regulated at weeks 4 and 8; and clusters 7 and 12, genes down-regulated at week 8. Cluster 5 genes were not associated with any significantly over-represented annotations, but this cluster of 27 genes (32 probe sets) included Cytl1 (cytokine-like 1), which has been shown to be expressed during chondrogenesis and to promote chondrocyte differentiation through stimulation of Sox9 transcriptional activity [15]. Cluster 10, also down-regulated at week 4, with continuing down-regulation in week 8, contained 11 genes. No functions were significantly over-represented in this cluster and none of these genes were associated with osteoarthritis in the literature. Cluster 10 contained a phosphatase, several kinases and four genes whose function involves nucleotide binding. Clusters 7 and 12, both down-regulated at week 8, consisted of 19 and 11 genes (21 and 11 probe sets), respectively. Significantly over-represented annotations identified by DAVID in cluster 7 included mitotic cell cycle, cell cycle process, cell division, and cytoskeleton. S100b, which had been used in the past as a marker for chondrogenesis [16] was in this cluster. Over-represented annotations in cluster 12 all involved carbohydrate metabolism.

Significant up-regulation of gene expression at 16 weeks was seen in clusters 15 and 16, both of which also exhibited high up-regulation at week 4. Cluster 15 consisted of 7 probe sets covering three genes; Prelp, Col3a1 (collagen, type III, alpha 1), and Fmod (fibromodulin). Prelp and fibromodulin were also present in the DMM/sham-Sham/baseline overlap gene list reported above, where they were found to be highly down-regulated at 16 weeks in the sham joints; however, these genes were highly up-regulated in the DMM joints. Cluster 16 was comprised of four probe sets, representing 3 genes: Col14a1 (collagen, type XIV, alpha 1), Bgn (biglycan), and Fbln7 (fibulin 7), all associated with the extracellular matrix. Col14a1 was identified in a microarray analysis of human tissue [17]; while Bgn was previously associated with chondrogenesis and extracellular matrix turnover in osteoarthritis [18].

Genes in clusters 3 and 11 displayed temporal profiles of up-regulation only at 4 weeks, with little significant change in expression from control at any other time point. Cluster 3 contained 45 genes (48 probe sets), with significant annotations that included regulation of transcription, DNA binding, regulation of RNA metabolic process, zinc ion binding, and transcription. Potential genes of interest were NFκB activating protein, Atf2 and Erg. Cluster 11 was composed of 11 genes, which exhibited no significantly over-represented functions. One of the genes in this cluster was Asb13, an ankyrin repeat and SOCS box-containing protein.

Clusters 1, 2, 4, 9, and 14 contained genes that exhibit strong up-regulation at 4 weeks, with less up-regulation at 2 weeks. Cluster 1 contained 48 genes (54 probe sets) representing genes whose significant annotations include embryonic organ morphogenesis, positive regulation of cell differentiation and developmental process. This cluster included several genes thought to be involved in joint biology and/or OA including syndecan 4, Bmp-1, and Timp-2. Cluster 2 contained 39 genes (52 probe sets) for genes also up-regulated at 2 and 4 weeks. The cluster included over-represented annotations for collagen, signal peptide, extracellular region, extracellular matrix, cell adhesion and glycosaminoglycan binding. Genes of potential interest in this cluster included collagen genes such as Col5a1 and Col16a1, growth factor-related genes such as Igf1 and Egfr, MMP-14, and biglycan. (Note, biglycan is represented by three probe sets on this mouse chip, two of which are found in cluster 16 and 1 in cluster 2). Clusters 4, 9, and 14 contained 33, 16 and 8 genes (33, 18, and 8 probe sets), respectively. DAVID did not identify any function annotations over-represented any of these clusters. Cluster 4 genes included two glutathione peroxidases, Gpx7 and Gpx8, perhaps suggesting some redox-related event.

Clusters 6, 8, and 13 also displayed the most up-regulation at week 4, but to a lesser extent than clusters 1, 2, 4, 9, and 14. Cluster 6 included 23 genes (24 probe sets) whose DAVID-identified over-represented annotations included signal peptide, glycoprotein, cell adhesion, serine/threonine kinase signaling pathway, skeletal system development and growth factor activity. Genes of particular interest included TGFβ2 and TGFβ3, latent TGFβ binding protein and Ddr2. Cluster 8 contained 19 genes for which the significant gene annotations included cell adhesion and contained COMP and Mmp13 which are thought to be important markers of the OA process. Cluster 13 included 9 genes for which there were no significantly over-represented functions. One of these, Enpp3, a pyrophosphatase, has been previously associated with osteoarthritis [19].

Overall, the analysis of gene clusters based on the metrics of both shape and magnitude of the time profile provided insight into the temporal process of osteoarthritis development. Only a few genes were down-regulated as part of this process, and those were down-regulated mainly at 4 and/or 8 weeks. Many more genes were up-regulated and most of the up-regulated genes displayed up-regulation at 4 weeks, with lesser up-regulation at 2 and 16 weeks. The phasic process was clearly visualized in these clusters, with the gene expression at 8 weeks being significantly different from gene expression at the other time points. This difference was consistent across all replicates.

### Tissue distribution of type III collagen, fibromodulin, and Prelp

**Figure 9:**
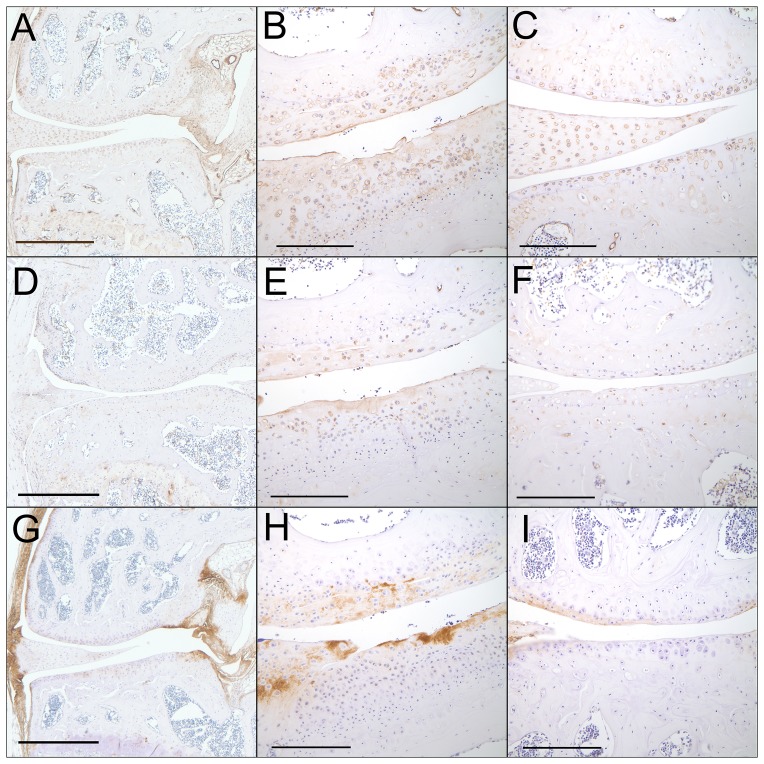
Immunolocalization of type III collagen, fibromodulin, and Prelp. Sections from mice in the 16 week-old sham and DMM groups were immunostained as detailed in the Methods using antibodies to type III collagen (A,B,C), fibromodulin (D,E,F) and Prelp (G,H,I). A, D, and G are 4x views of sham control joints to demonstrate overall tissue distribution. B, E, and H are 20x views of DMM joints and C, F, and I are 20x views of sham control joints. Bar  = 1 mm for 4X, 200um for 20X.

Based on the results of the consensus clustering, we choose to immunostain for type III collagen, fibromodulin, and Prelp, in order to determine which tissues might be providing the signals noted by gene expression at week 16. A total of 3 DMM and 4 sham control mice were studied and representative images are shown in **Figure 9** The tissue distribution of immunopositivity was similar between the two surgical groups; however, certain tissue types (e.g. fibrocartilage) were more prevalent in the DMM mice. Type III collagen immunopositivity was present in a pericellular distribution in articular cartilage and the meniscus and was located more diffusely in ligaments, the joint capsule, and the synovium as well as in the fibrocartilage located over osteophytes (**Table 3**). It was also present in the vascular endothelium. Fibromodulin immunopositivity was present in low numbers of articular chondrocytes, minimally in meniscus, and variably in synovium. Immunopositivity was more intense and involved more chondrocytes in fibrocartilage, notably over osteophytes, than in articular cartilage. Some bone marrow cells, including megakaryocytes also were positive. Immunopositivity for Prelp was present in ligaments (particularly at the site of ligamentous attachments to bone), periosteum, and fibrous connective tissue. Most chondrocytes were negative; however the matrix of degenerative cartilage and fibrocartilage was strongly immunopositive.

**Table 3:** Immunostaining results for Prelp, Type III collagen, and Fibromodulin.

Tissue	Prelp	Type III collagen	Fibromodulin
Ligaments	+++/++++	+	+/−
Periosteum	+++	+	+/−
Fibrous connective tissue	++	+	−
Articular cartilage chondrocytes	−	−	+/−
Articular cartilage matrix	+/−	++ (mainly pericellular matrix)	−
Meniscus chondrocytes	+/−	+	+/−
Meniscus matrix	+/−−	++ (mainly pericellular matrix)	+
Bone	−	−	−
Bone marrow	−	−	++
Growth plate chondrocytes	+/−	−	−
Growth plate matrix	+/−	+/−	+
Fibrocartilage	++	++ (mainly pericellular matrix of chondrocytes)	++
Skeletal muscle	−	+ (perimysium only)	+/−
Synovium	−	+++	+/++
Degenerate/fibrillated cartilage	++/+++	++	++
Vascular endothelium	−	++++ (also vessel wall)	−

Prelp =  proline-arginine-rich end leucine-rich repeat protein. Sections from 3 DMM and 4 sham control animals at 16 weeks after surgery were graded on a scale from no immunopositivity (−) to marked immunopositivity (++++). The results presented are the consensus grades from all animals evaluated. The degenerate/fibrillated cartilage was only present in the DMM animals.

### Real-time PCR results for selected genes

We selected a set of 16 genes to evaluate using RT-PCR based on genes tested in our previous study [6] where we had compared RT-PCR results at the 8 week time point in young and older adult mice. The average fold change in the DMM relative to sham controls for this gene set was highest at 4 weeks and lowest at 8 weeks (**Table 4**) which was consistent with the overall pattern of the 371 genes found to be significantly regulated in DMM vs sham joints on the microarrays (**Figure 6**). Genes with significant upregulation at 4 weeks included aggrecan, asporin, Ccl21, Cxcr7, Dkk3, Htra1, Igf-1, periostin, and Sfrp2. Looking across the time course, none of the genes examined were significantly up- or down-regulated in DMM relative to sham joints at all 4 time points; however, Ccl21, Cxcr7, Igf-1, and Sfrp2 were up-regulated at 3 of 4 time points. The two Wnt pathway regulators that were examined, Dkk3 and Sfrp2, were both significantly up-regulated at the early time points as was Col3a1. Unlike Dkk3 and Sfrp2, Col3a1 was also highly expressed at 16 weeks, consistent with the microarray results. Although type X collagen has been used as a marker of chondrocyte hypertrophy, which is thought to contribute to the development of OA, we actually observed a decrease in its expression at two weeks and no significant increase at the later time points. MMP-13 was increased at 2, 4, and 16 weeks in DMM relative to joints, as was noted on the microarrays, but due to variability among the 3 pools it did not reach statistical significance.

**Table 4:** Real time PCR results for selected genes showing fold change in DMM joints relative to sham controls.

Time point	2 weeks		4 weeks		8 weeks		16 weeks	
Gene name	fold change	p value	fold change	p value	fold change	p value	fold change	p value
Adamts5	0.37	NS	0.26	NS	0.86	NS	0.96	NS
Aggrecan	2.53	NS	5.90	0.006	1.06	NS	2.00	NS
Asporin	1.45	NS	2.50	0.04	1.60	0.05	2.00	NS
Ccl21	1.48	NS	2.41	0.001	2.27	0.003	1.57	0.05
Ccr7	0.48	0.006	1.00	NS	1.83	0.05	1.00	NS
Cxcr7	2.43	NS	2.77	0.03	1.51	0.02	2.99	0.03
Col3	2.24	0.05	4.28	NS	1.93	NS	3.34	0.004
Col10	0.48	0.02	0.25	NS	1.75	NS	1.17	NS
Dkk 3	4.27	0.01	2.50	0.02	1.77	NS	2.50	NS
Htra1	1.39	NS	4.09	0.01	1.81	0.008	3.00	NS
Igf-1	1.88	NS	3.50	0.0004	3.00	0.002	3.00	0.008
IL33	1.50	NS	1.50	NS	1.61	0.05	1.88	NS
Mmp3	3.68	0.03	10.57	NS	2.32	0.007	2.22	NS
Mmp13	1.48	NS	2.00	NS	1.08	NS	1.46	NS
Periostin	4.48	NS	3.38	0.009	1.44	NS	1.75	NS
Sfrp2	2.92	0.01	2.81	0.002	1.56	0.01	1.29	NS
Average fold change	2.07		3.11		1.71		2.01	

## Discussion

Both the histologic and gene expression analysis support a phasic process for the development of OA in this model. The early phases at 2 and 4 weeks after DMM surgery were the most active in terms of gene expression at a time when the earliest cartilage lesions in the medial tibial plateau were very mild but abaxial chondrophytes had already formed. The chondrophytes matured to osteophytes as the articular cartilage lesions became more severe but, interestingly, this was accompanied by a significant decline in overall gene expression at the 8 week time point. In addition to the differences in overall gene expression, the findings from cluster analysis showed that each time point studied had a unique gene signature that also supports a phasic process.

Perhaps most striking was the decline in gene expression at 8 weeks after surgery. There was a significant down-regulation of genes regulating cell proliferation while matrix remodeling genes that were up-regulated at 2 and 4 weeks were mostly off, suggesting that a quiescent phase had been reached. However, at 16 weeks this pattern had changed and was accompanied by significant cartilage loss in the medial tibial plateau while cartilage damage and chondrocyte death was beginning to be evident in the lateral tibial plateau along with the appearance of lateral axial osteophytes. Because RNA for the gene arrays was only isolated from the medial side of the joint, the changes in gene expression observed at 16 weeks were not due to the disease progressing laterally but rather reflect more advanced disease medially. The lack of time points between 4 and 8 weeks and 8 and 16 weeks prevents any conclusions about the length of the quiescent phase.

The significant expression of Prelp, Col3a1, and fibromodulin at 16 weeks suggests that a matrix repair or a “wound healing response” was active. At 16 weeks, the articular cartilage from the DMM group had severe lesions in the medial tibial plateau with some loss of cartilage. This suggested that the signal at 16 weeks for these genes may have come from either early changes on the femoral side, since some femoral tissue was included for RNA extraction, or more likely, from other joint structures such as the meniscus, synovium, ligaments and subchondral bone. Immunostaining revealed that ligaments and the fibrocartilage found over osteophytes were common locations for all three proteins. These results are consistent with previous studies that examined the location and function of these proteins. Prelp is thought to play a role in matrix organization through interactions with collagen, as does the small leucine-rich proteoglycan fibromodulin [20], [21]. Deletion of fibromodulin in mice leads to OA, possibly via weakened ligaments and tendons [22]. However, a recent study that revealed a role for complement activation in OA demonstrated increased levels of fibromodulin in OA synovial fluid and found that fibromodulin could promote the activation of complement [23]. Overexpression of Prelp has been shown to disrupt normal collagen fiber formation in skin [24] and, in contrast to fibromodulin, it is an inhibitor of complement activation [25]. Thus, too little or too much fibromodulin and Prelp could contribute to OA.

Increased expression of type III collagen has been previously noted in human OA cartilage [26], [27] and it has been found in normal cartilage in fibrils containing type II collagen [28]. We noted a pericellular location for type III collagen in articular cartilage and the meniscus. It is not known what role type III collagen could have in the OA joint. In the set of 371 genes that were differentially expressed in DMM vs sham joints, we observed increased expression, at one or more time points, in a large number of collagen genes that are likely part of an attempted repair response. These included the alpha 1 chain of types III, V, XII, XIV, XVI, and XXII collagen and the alpha 2 chain of type IV collagen, along with the collagen processing genes, procollagen c-endopeptidase enhancer protein and procollagen-lysine, 2-oxoglutarate 5-dioxygenase 1.

The phasic changes in gene expression noted in the DMM knees were not the result of aging of the animals over the 16 week time course. Changes in gene expression were noted when we compared the 12 week old animals at baseline (un-operated) and the sham controls over the 16 week time course but these differed significantly from the changes in gene expression noted in the DMM relative to sham joints. This was seen in the overlap analysis where the changes over time in expression in the DMM joints were most often in the opposite direction to changes over time in the sham joints. This was most evident for Prelp and fibromodulin which were significantly down-regulated at week 16 in the sham controls but increased in the DMM joints. Interestingly, immunopositivity for both of these proteins was notably stronger and more extensive in fibrocartilage over osteophytes than in articular or meniscal cartilage. In addition, immunopositivity for Prelp was increased in degenerative cartilage matrix compared with normal cartilage. In the sham time course analysis, most genes exhibited an up-regulation of expression at 2 weeks followed by a progressive decline out to 16 weeks, compared to time zero. Many of these genes were involved in morphogenesis and development, suggesting the animals were more actively growing at the start of the study and then growth likely slowed. Importantly, the progressive increase in subchondral bone area and thickness were the only histologic measures that changed with time in all groups (control, sham and DMM).

We have recently reported a comparison of the 8 week time point results included in the present study with results from the same time point in animals that were 12 months-old at the time of DMM surgery [6]. The histologic OA severity in the older mice was about twice that of the younger mice and significantly more genes were up-regulated in the older mice. The 8 week comparisons suggest that the older mice develop OA more rapidly after DMM surgery than the younger mice, a result which could have contributed to the differences in gene expression at 8 weeks. A major difference between the young and older mice was in the expression of muscle related genes which were up-regulated in the older mice and either down-regulated or unchanged in the younger mice. Consistent with greater importance of the muscle-related genes in the older mice, we did not find significant annotations for muscle in the time course study using young mice. It would be of interest to do a time course study in the 12 month-old mice to determine if a phasic progression is also found. We did compare the DMM vs sham gene expression from the 8 week time point in the 12 month old mice to the other time points in the younger mice and found the most genes in common at 16 weeks (158 genes) suggesting more rapid progression in the older mice (data not shown).

A phasic progression of OA was suggested by a previous study in humans where radiographic progression was related to changes in serial measures of serum COMP, as an OA biomarker [29]. Interestingly, we found that COMP, along with MMP-13, clustered with genes that were up-regulated during times of increased activity at 2, 4, and 16 weeks and were down-regulated at 8 weeks when activity was lowest. One potential explanation for a phasic process would be the maturation of osteophytes which have been proposed to stabilize the joint mechanics [30]. Our observation that chondrophytes had already formed by 2 weeks after surgery to destabilize the meniscus is consistent with the hypothesis that joint destabilization stimulates their formation. Increased expression of Bmp-1, TGFβ2 and TGFβ3 at the early time points is also consistent with their presumed role in stimulating osteophyte formation [31]. The stabilization, however, appeared to be transient because cartilage loss was marked at 16 weeks and gene expression was increased relative to 8 weeks.

There are important limitations to the present study. Because RNA was not isolated from individual tissues it is not possible to determine which specific tissue (cartilage, bone, meniscus, ligament, synovium) contributed to the changes in gene expression. Also, if a gene is expressed in a single tissue, pooling of tissues will reduce the signal to a lower level than what would have been observed in the individual tissue. That is the likely explanation for the overall lower levels of gene expression observed in the present study when compared to work using a single tissue. Based on the immunostaining for type III collagen, fibromodulin, and Prelp, as well as immunostaining for IL-33, periostin, and CCL21 reported in our previous study [6], it appears that multiple joint tissues can be responsible for changes in gene expression consistent with the joint operating as an organ rather than as individual tissues. Therapeutic interventions for OA which target key processes occurring in multiple joint tissues rather than a single tissue should be more effective than those targeting a single tissue. Another limitation is that the DMM model may or may not reflect human OA disease pathogenesis. Certainly the histologic features in cartilage and bone are similar, but in human OA there may be more disease activity in the synovium than in the DMM model. Finally, we studied gene expression, and not protein production, and these do not always correlate. Immunohistochemical studies to assess changes at the protein level are not sensitive enough to detect differences noted at the level of gene expression, unless either the differences in expression are marked, or the protein being examined is only detected in diseased tissue and not in normal tissue.

Further studies will be needed to determine the importance of these findings for human OA. An important implication of the results is that the degree of response to an intervention given during a phasic process will depend on the timing of the intervention. Pre-clinical studies using animal models most often start the intervention at the same time or just after the start of the OA process while in human trials participants are likely to be at various stages of the disease process when the intervention is initiated. Finding markers of various disease stages in OA could be used to direct targeted therapy to the proper phase of the disease when the target is most active.

## Supporting Information

Table S1 **Filtering results for the sham time course.**
(DOCX)

Table S2 **Filtering results for the DMM time course.**
(DOCX)

File S1 **Excel file of 406 Sham filtered probe sets and DAVID results.** Results are presented as signal log ratio (SLR) which is the log_2_ of the fold change and so an SLR of 0.5 =  1.4-fold. A positive number would be up-regulation and negative number down-regulation.
(XLSX)

File S2 **Excel file of 427 DMM filtered probe sets and DAVID results.** Results are presented as signal log ratio as described for supplemental file 1.
(XLSX)

File S3 **Excel file of 97 common probe sets and DAVID results.**
(XLSX)
