# Supplementary material for: Disease Progression and Phasic Changes in Gene Expression in a Mouse Model of Osteoarthritis
Source: PLoS One. 2013 Jan 28;8(1):e54633. doi: 10.1371/journal.pone.0054633 (PMC3557277; doi:10.1371/journal.pone.0054633)
Supplement: Table S1 — Filtering results for the sham time course. (DOCX) [file pone.0054633.s001.docx]

## Table S1: Filtering results for the sham time course.

| ***Replicate*** | ***Total Transcripts on Chip*** | ***Detection***  ***P-value Filter*** | ***SLR Filter*** | ***Overlap Analysis*** | ***PCC and ED Filter*** |
| --- | --- | --- | --- | --- | --- |
| Rep1 | 45,101 | 22,601 | 2,707 | 1,183 | 406 |
| Rep2 |  | 23,338 | 2,439 |  |  |
| Rep3 |  | 23,154 | 4,276 |  |  |
