# Supplementary material for: Disease Progression and Phasic Changes in Gene Expression in a Mouse Model of Osteoarthritis
Source: PLoS One. 2013 Jan 28;8(1):e54633. doi: 10.1371/journal.pone.0054633 (PMC3557277; doi:10.1371/journal.pone.0054633)
Supplement: Table S2 — Filtering results for the DMM time course. (DOCX) [file pone.0054633.s002.docx]

## Table S2: Filtering results for the DMM time course.

| ***Replicate*** | ***Total Transcripts***  ***on Chip*** | ***Detection***  ***P-value Filter*** | ***SLR Filter*** | ***Overlap Analysis*** | ***PCC and ED Filter*** |
| --- | --- | --- | --- | --- | --- |
| Rep1 | 45,101 | 23,055 | 2,130 | 1,101 | 427 |
| Rep2 |  | 23,059 | 3,846 |  |  |
| Rep3 |  | 22,822 | 2,740 |  |  |
